# Composite Measure of Physiological Dysregulation as a Predictor of Mortality: The Long Life Family Study

**DOI:** 10.3389/fpubh.2020.00056

**Published:** 2020-03-06

**Authors:** Konstantin G. Arbeev, Olivia Bagley, Svetlana V. Ukraintseva, Hongzhe Duan, Alexander M. Kulminski, Eric Stallard, Deqing Wu, Kaare Christensen, Mary F. Feitosa, Bharat Thyagarajan, Joseph M. Zmuda, Anatoliy I. Yashin

**Affiliations:** ^1^Biodemography of Aging Research Unit, Social Science Research Institute, Duke University, Durham, NC, United States; ^2^Danish Aging Research Center, Department of Public Health, University of Southern Denmark, Odense, Denmark; ^3^Division of Statistical Genomics, Department of Genetics, Washington University School of Medicine, St. Louis, MO, United States; ^4^Department of Laboratory Medicine and Pathology, University of Minnesota, Minneapolis, MN, United States; ^5^Department of Epidemiology, University of Pittsburgh, Pittsburgh, PA, United States

**Keywords:** physiological dysregulation, statistical distance, mortality, prediction, Long Life Family Study, deficits index, aging

## Abstract

Biological aging results in changes in an organism that accumulate over age in a complex fashion across different regulatory systems, and their cumulative effect manifests in increased physiological dysregulation (PD) and declining robustness and resilience that increase risks of health disorders and death. Several composite measures involving multiple biomarkers that capture complex effects of aging have been proposed. We applied one such approach, the Mahalanobis distance (D_M_), to baseline measurements of various biomarkers (inflammation, hematological, diabetes-associated, lipids, endocrine, renal) in 3,279 participants from the Long Life Family Study (LLFS) with complete biomarker data. We used D_M_ to estimate the level of PD by summarizing information about multiple deviations of biomarkers from specified “norms” in the reference population (here, LLFS participants younger than 60 years at baseline). An increase in D_M_ was associated with significantly higher mortality risk (hazard ratio per standard deviation of D_M_: 1.42; 95% confidence interval: [1.3, 1.54]), even after adjustment for a composite measure summarizing 85 health-related deficits (disabilities, diseases, less severe symptoms), age, and other covariates. Such composite measures significantly improved mortality predictions especially in the subsample of participants from families enriched for exceptional longevity (the areas under the receiver operating characteristic curves are 0.88 vs. 0.85, in models with and without the composite measures, *p* = 2.9 × 10^−5^). Sensitivity analyses confirmed that our conclusions are not sensitive to different aspects of computational procedures. Our findings provide the first evidence of association of PD with mortality and its predictive performance in a unique sample selected for exceptional familial longevity.

## Introduction

Traditional demographic analyses based on information from population life tables provide useful insights on historical patterns of change in mortality, survival curves, and life expectancy which can also be used to predict future trends in these characteristics in the entire population or specific subpopulations. However, such “aggregated” predictions provide information for an “average” individual from a (sub)population and may yield little information about mortality risk and remaining life expectancy for some individuals which are determined by their unique histories of exposures to various risk factors during the life-course, by their genetic makeup, or the interaction of these risk factors and genetics. Therefore, although age is an important risk factor for mortality and determinant of remaining life expectancy, individuals of the same age can have very diverse and unique characteristics that affect their current health status and future risks of health deterioration and mortality. Measurements of different physiological and other variables (biomarkers) provide additional opportunity for personalized predictions of morbidity and mortality risks as they can reflect individual age-related changes occurring at the molecular and cellular levels in different organs and tissues that result in individual-specific rates of physiological dysregulation, health deterioration, and mortality risks. Composite measures based on multiple biomarkers of different physiological systems [see e.g., (1–4) and recent reviews (5, 6)] can capture the complex effect of aging on different regulatory systems and its relation to morbidity and mortality.

Recently, the statistical (Mahalanobis) distance (denoted as D_M_) constructed for the joint distribution of multiple biomarkers was suggested as a composite measure that can represent the level of physiological dysregulation in an organism (2) and aging-related declines in robustness and resilience (7). This measure was associated with mortality and aging-related outcomes in numerous studies [see e.g., (2, 7–12)]. In this paper, we constructed D_M_ using measurements of multiple biomarkers collected at the baseline visit of the Long Life Family Study (LLFS) to test if the level of PD is associated with mortality in this study, and whether it improves mortality predictions compared to the models with age and common individual risk factors. The LLFS is a unique study which enrolled participants from families selected for exceptional familial longevity (13), along with their spouses. The LLFS participants from the probands' generation have much better survival chances than their age peers from the general population so that the survival curves for the LLFS participants are shifted to the right compared to population survival functions computed from respective cohort life tables (14). Hence, unlike all previous studies applying D_M_, this paper investigates whether D_M_ is a useful predictor of mortality in persons with much lower mortality risk compared to a general population (and who, respectively, have much higher remaining life expectancy than that estimated from population-based cohort life-tables).

## Materials and Methods

### Data

The LLFS is a family-based, longitudinal study of healthy aging and longevity that enrolled participants at field centers in the US (Boston, New York, Pittsburgh) and Denmark. During the baseline visit in 2006–2009, more than 4,900 participants were enrolled from families determined to have exceptional longevity according to the Family Longevity Selection Score (FLoSS) (13). Details on study eligibility criteria are described elsewhere (15). Socio-demographic variables, data on past medical history and current medical conditions, medications use, physical and cognitive functioning, and blood samples were collected via in-person visits and phone questionnaires for all subjects at the time of enrollment (15). Blood assays were centrally processed at a Laboratory Core (University of Minnesota) and protocols were standardized, monitored and coordinated through a Data Management Coordinating Center (Washington University, St. Louis). Written informed consent was obtained from all subjects following protocols approved by the respective field center's Institutional Review Boards (IRBs). In this paper, we performed secondary analyses of LLFS data collected at all field centers. This study was approved by the Duke Health IRB.

LLFS participants were followed up annually to track their vital and health status. The analyses reported in this paper used the March 11, 2019 release of LLFS data with the latest recorded date of death on February 13, 2019. Ages at the baseline visit were validated using dates of birth from official documents (such as birth certificate or driver's license) (16). Ages at death were computed from available dates of birth and death. Ages at censoring for those who did not die within the follow-up period were determined from dates of birth and the last follow-up in the March 11, 2019 release of LLFS data. We also computed prevalence (i.e., the disease status at the baseline) and incidence (i.e., new cases reported during the follow-up) of major diseases available in the study such as Alzheimer's disease (AD) or dementia, cancer, cardiovascular diseases (CVD), diabetes, for the entire sample and for the reference population used in construction of D_M_ [see section Construction of the Cumulative Measure of Physiological Dysregulation (D_M_)]. Information on health conditions was collected during the interviews from study participants or proxies (if a participant was unable to provide an answer). At the baseline, the question asked was “Please respond ‘yes’ or ‘no’ if you have EVER been told by a doctor that you had this condition.” Similar questions were asked during follow-up interviews (“Please respond ‘yes’ or ‘no’ if you have EVER been told by a doctor that you had this condition since we last interviewed you on…”). Using responses to such questions about specific diseases [AD or dementia: Alzheimer's Disease or Dementia; cancer: All cancer cites; CVD: Myocardial Infarction, Heart Attack, Coronary Angioplasty, Coronary Artery, Bypass Grafting, (Congestive) Heart Failure, Stroke, Cerebrovascular Accident, Transient Ischemic Attack, or Mini-Stroke; diabetes: Diabetes] from the baseline and follow-up interview, we computed the numbers of prevalent cases at the baseline and the numbers of new cases reported since the baseline.

In addition to association and predictive analyses with D_M_ described in section Association of D_M_ with Mortality and Predictive Performance Analyses, we conducted descriptive analyses of the original variables as well as D_M_ in the entire sample and in generation (probands' and offspring) and spouse groups (probands, their siblings and offspring, and their spouses). Specifically, we computed empirical characteristics (means, standard deviations, ranges, correlations with age, percentages) for various variables used in the analyses (see Table 1). Relevant tests (one-way ANOVA, *t*-test, chi-square test) were used to provide statistical inference (see section Descriptive Analyses). We also computed the Kaplan-Meier estimates of survival curves (conditional at age 80 years) for subsamples of participants grouped by the quartiles of the distribution of D_M_ in the analyzed sample. Age at the baseline or age 80 years (whatever was the largest) was used as the left truncation variable for these analyses. The quartiles were computed separately by sex and in the entire sample as reported in respective figures (see section Descriptive Analyses). The 95% confidence intervals for the survival curves were computed based on respective estimates of cumulative hazards.

**Table 1:** Sample characteristics of the Long Life Family Study at the baseline visit.

**Sample characteristics**	**Probands' generation**		**Offspring generation**		**Total sample**
	**Probands and their siblings**	**Spouses**	**Offspring**	**Spouses**	
Number of participants at baseline	1,500	191	2,435	812	4,938
Number of deaths during the follow-up period	1,174	103	178	70	1,525
Age at baseline^*^	90.4 ± 6.4 [49, 110]	83.4 ± 7.0 [55, 101]	60.5 ± 8.3 [30, 88]	60.9 ± 8.7 [24, 88]	70.5 ± 15.8 [24, 110]
Females (%)	52.67	78.01	57.49	47.41	55.16
Whites (%)	98.93	98.95	99.59	98.65	99.21
Participants from US field centers (%)	84.6	84.82	74.25	53.08	74.32
Low educated participants (below high school) (%)	25.87	17.28	5.79	9.11	12.88
Smokers (smoked > 100 cigarettes in lifetime) (%)	37.33	39.27	45.34	45.69	42.73
Medication use: anti-diabetic (%)	6.93	7.85	4.6	4.8	5.47
Medication use: anti-hypertensive (%)	66.87	71.73	30.14	35.1	43.72
Medication use: lipid-lowering (%)	30.33	42.41	25.09	25.74	27.46
Fasting (≥8 h) (%)	87.8	91.1	88.79	92.73	89.23
Follow-up period^*^	5.8 ± 3.2 [0, 12.3]	7.0 ± 3.3 [0, 12.0]	9.6 ± 2.3 [0, 12.5]	9.3 ± 2.7 [0, 12.3]	8.3 ± 3.2 [0, 12.5]
Follow-up period for dead^*^	4.8 ± 2.7 [0, 11.9]	5.3 ± 2.7 [1, 11.1]	6.3 ± 2.8 [1, 12.4]	6.2 ± 2.6 [1, 11.3]	5.1 ± 2.8 [0, 12.4]
Follow-up period for alive^*^	9.4 ± 2.4 [0, 12.3]	9.0 ± 2.7 [0, 12.0]	9.9 ± 2.1 [0, 12.5]	9.6 ± 2.5 [0, 12.3]	9.8 ± 2.2 [0, 12.5]
Prevalence of cancer, *N* (%)	529 (35.27)	60 (31.41)	430 (17.66)	161 (19.83)	1,180 (23.90)
Prevalence of CVD, *N* (%)	419 (27.93)	43 (22.51)	134 (5.50)	68 (8.37)	664 (13.45)
Prevalence of AD or dementia, *N* (%)	111 (7.40)	9 (4.71)	2 (0.08)	6 (0.74)	128 (2.59)
Prevalence of diabetes, *N* (%)	136 (9.07)	22 (11.52)	145 (5.95)	54 (6.65)	357 (7.23)
Incidence of cancer, *N* (%)	158 (10.53)	26 (13.61)	274 (11.25)	98 (12.07)	556 (11.26)
Incidence of CVD, *N* (%)	310 (20.67)	42 (21.99)	141 (5.79)	61 (7.51)	554 (11.22)
Incidence of AD or dementia, *N* (%)	113 (7.53)	15 (7.85)	21 (0.86)	9 (1.11)	158 (3.20)
Incidence of diabetes, *N* (%)	21 (1.40)	2 (1.05)	94 (3.86)	32 (3.94)	149 (3.02)
Adiponectin (Adip)^**^	16,084.1 ± 9,791 0.15 [3.8 × 10^−8^]	15,369.9 ± 7,812 0.14 [0.07]	10,967.1 ± 6,459 0.10 [1.8 × 10^−6^]	10,198.8 ± 5,800 0.04 [0.28]	12,526.9 ± 7,959 0.32 [0.0]
Albumin (Album)^**^	3.8 ± 0.3 −0.20 [2.1 × 10^−14^]	3.9 ± 0.3 −0.26 [5.3 × 10^−4^]	4.1 ± 0.3 −0.08 [4.4 × 10^−5^]	4.1 ± 0.3 −0.11 [1.8 × 10^−3^]	4.0 ± 0.3 −0.36 [0.0]
Absolute monocyte count (Abs.M)^**^	0.7 ± 0.3 0.11 [5.0 × 10^−5^]	0.7 ± 0.4 −3.8 × 10^−4^ [1.00]	0.6 ± 0.2 0.11 [5.6 × 10^−7^]	0.6 ± 0.4 0.09 [0.02]	0.6 ± 0.3 0.20 [0.0]
Creatinine (Creat)^**^	1.2 ± 0.4 0.11 [3.1 × 10^−5^]	± 0.3 0.14 [0.07]	± 0.3 0.10 [2.4 × 10^−6^]	± 0.2 0.23 [5.2 × 10^−11^]	± 0.3 0.31 [0.0]
Cystatin (Cysc)^**^	1.5 ± 0.5 0.32 [0.0]	1.2 ± 0.4 0.35 [1.8 × 10^−6^]	0.9 ± 0.3 0.33 [0.0]	0.9 ± 0.2 0.44 [0.0]	± 0.4 0.63 [0.0]
Dehydroepiandrosterone sulfate (DHEA)^**^	44.6 ± 30.9 −0.12 [1.7 × 10^−4^]	42.3 ± 30.9 −0.28 [9.8 × 10^−4^]	91.0 ± 63.6 −0.30 [0.0]	95.2 ± 65.5 −0.27 [2.6 × 10^−13^]	78.3 ± 60.6 −0.43 [0.0]
Hemoglobin (Hgb)^**^	13.2 ± 1.4 −0.22 [0.0]	13.5 ± 1.4 −0.20 [9.5 × 10^−3^]	14.2 ± 1.3 −0.08 [2.1 × 10^−4^]	14.3 ± 1.2 0.08 [0.03]	13.9 ± 1.4 −0.34 [0.0]
Glycosylated hemoglobin (HbA1c)^**^	5.8 ± 0.5 0.03 [0.22]	5.8 ± 0.7 −0.02 [0.78]	5.6 ± 0.6 0.21 [0.0]	5.6 ± 0.5 0.18 [4.8 × 10^−7^]	5.6 ± 0.6 0.22 [0.0]
High-sensitivity C-reactive protein (hsCRP)^**^	5.1 ± 11.5 0.09 [7.3 × 10^−4^]	5.0 ± 11.8 0.03 [0.65]	2.7 ± 4.4 0.03 [0.12]	2.8 ± 6.6 0.04 [0.26]	3.5 ± 7.9 0.15 [0.0]
Insulin-like growth factor 1 (IGF1)^**^	103.3 ± 47.2 −0.24 [0.0]	104.3 ± 42.8−0.28 [2.4 × 10^−4^]	144.1 ± 170.7 −0.02 [0.28]	140.9 ± 49.9−0.18 [6.7 × 10^−7^]	129.9 ± 126.3 −0.16 [0.0]
Interleukin 6 (IL-6)^**^	4.3 ± 10.0 0.13 [2.3 × 10^−6^]	2.7 ± 4.4 0.12 [0.10]	1.4 ± 3.6 0.04 [0.05]	1.5 ± 4.4 0.11 [2.2 × 10^−3^]	2.3 ± 6.5 0.21 [0.0]
Mean corpuscular volume (MCV)^**^	93.8 ± 6.1 −4.8 × 10^−4^ [0.99]	94.6 ± 5.6 0.08 [0.27]	92.0 ± 5.2 0.07 [1.3 × 10^−3^]	91.6 ± 5.2 0.14 [1.0 × 10^−4^]	92.6 ± 5.6 0.17 [0.0]
N-terminal pro b-type natriuretic peptide (NT-proBNP)^**^	899.8 ± 1588.1 0.18 [9.6 × 10^−12^]	487.9 ± 614.0 0.31 [3.2 × 10^−5^]	105.4 ± 272.1 0.19 [0.0]	99.6 ± 160.5 0.28 [5.3 × 10^−15^]	359.5 ± 974.8 0.38 [0.0]
Red cell distribution width % (RDW)^**^	14.5 ± 1.5 0.15 [3.1 × 10^−8^]	14.3 ± 1.1 0.23 [1.8 × 10^−3^]	13.6 ± 1.1 0.12 [3.2 × 10^−8^]	13.7 ± 1.2 0.18 [3.3 × 10^−7^]	13.9 ± 1.3 0.31 [0.0]
Sex-hormone binding globulin (SHBG)^**^	85.4 ± 39.1 0.16 [6.3 × 10^−10^]	80.1 ± 34.2 0.23 [2.4 × 10^−3^]	61.2 ± 36.0 0.02 [0.29]	59.9 ± 32.1 −0.09 [0.01]	69.0 ± 38.0 0.28 [0.0]
Soluble receptor for advanced glycation endproduct (sRAGE)^**^	822.1 ± 626.1 0.14 [2.9 × 10^−7^]	699.6 ± 405.3 0.16 [0.03]	539.8 ± 401.6 0.06 [1.9 × 10^−3^]	532.4 ± 430.3 0.02 [0.67]	629.0 ± 501.1 0.26 [0.0]
Total cholesterol (T.Chol)^**^	187.3 ± 43.7 −0.03 [0.26]	199.3 ± 45.8 −0.20 [6.9 × 10^−3^]	205.5 ± 40.0 −0.01 [0.49]	204.8 ± 40.1 −0.05 [0.13]	199.7 ± 42.2 −0.18 [0.0]
Transferrin receptor (Transf.R)^**^	3.3 ± 1.2 0.06 [0.02]	3.1 ± 1.0 0.05 [0.55]	2.9 ± 1.1 9.8 × 10^−3^ [0.64]	2.9 ± 1.4 0.06 [0.10]	3.0 ± 1.2 0.14 [0.0]
White blood cell count (WBC)^**^	6.8 ± 2.9 0.07 [0.01]	6.8 ± 2.9 −0.02 [0.84]	6.0 ± 1.8 0.09 [5.2 × 10^−5^]	6.0 ± 1.7 0.14 [6.6 × 10^−5^]	6.3 ± 2.2 0.19 [0.0]

(1) * These rows display mean ± SD [range is shown in brackets];

(2) ** *these rows display mean ± SD and correlation with age [p-value for the null hypothesis on zero correlation is shown in brackets]; (3) Number of missing data: education−14; smoking−23; anti-diabetic drugs−498; anti-hypertensive drugs−498; lipid-lowering drugs−498; fasting−19; Adip−453; Album−262; Abs.M−408; Creat−245; Cysc−262; DHEA−993; Hgb−383; HbA1c−277; hsCRP−455; IGF1–466; IL-6–310; MCV−384; NT-proBNP−320; RDW−397; SHBG−263; sRAGE−261; T.Chol−245; Transf.R−453; WBC−382; Other variables listed in the table have no missing values. (4) The numbers shown in “Number of deaths during the follow-up period” and “Follow-up period” correspond to the LLFS data release used in this paper (see section Data)*.

### Construction of the Cumulative Measure of Physiological Dysregulation (D_M_)

The statistical (Mahalanobis) distance (17, 18) constructed for the joint distribution of biomarkers was recently suggested in the literature as an approach to construct a composite measure (denoted as D_M_) that reflects physiological dysregulation in aging body (2, 9, 11). It is designed to measure how “aberrant each individual's profile is with respect to the overall average (centroid) of the reference population” (10) that represents the “normal” physiological state. Such “reference population” can be either a subsample of the same study or it can come from an external study. Here we constructed D_M_ using baseline observations of 19 biomarkers that were used in the study of biomarker signatures of aging in LLFS (19). The list of biomarkers (that includes inflammation, hematological, diabetes-associated, lipid, endocrine, and renal biomarkers) along with their descriptive statistics (means, standard deviations, correlation with age) is presented in Table 1. The initial sample contained 4,938 individuals participating in LLFS visit 1. The notes under Table 1 contain information about numbers of missing observations of these biomarkers and other variables used in the analyses. After exclusion of individuals with at least one missing value of respective variables, the resulting sample used in construction and analyses of D_M_ included 3,279 participants (1,815 females, 1,464 males, 886 probands/siblings, 128 spouses of probands/siblings, 1,691 offspring, and 574 offspring spouses). Further, in the Cox regression analyses described below, we removed 19 individuals that were lost to follow-up right after visit 1 (their age at censoring was set to age at baseline) so that the resulting sample size for the Cox regression model was 3,260 individuals. See also section Sensitivity Analyses regarding analyses using multiple imputation of missing values.

Observed values of each biomarker were transformed using the Box-Cox transformation and standardized so that the transformed biomarkers are all on the same scale (with a zero mean and a unit variance). When a variable had zeros for some individuals, all records for that variable were shifted by adding 0.1, so that the Box-Cox transformation could work. We used individuals younger than 60 years at the baseline as a “reference population.” This cutoff age produced a reasonably large “reference population” for the current analyses (1,361; 815 females, 546 males). Computations of the means and variance-covariance matrix in the “reference population” [which are needed for construction of D_M_ (2)] were performed separately for females and males. The resulting D_M_ was also transformed using the Box-Cox transformation (see also description of additional computations in section Sensitivity Analyses). Table S1 provides characteristics of the reference population used for construction of D_M_.

### Association of D_M_ With Mortality and Predictive Performance Analyses

We fitted the Cox proportional hazards models with adjustment for related individuals (sandwich estimator) to follow-up data on mortality in the entire LLFS sample. We also performed analyses stratified by generation (probands' generation and offspring generation) and spouse status (probands, their siblings and offspring, and their spouses). Age was used as a time variable with age at visit 1 included as the left truncation variable in the model. The models were adjusted for the following covariates (in addition to D_M_): sex (1—male, 0—female), field center (four levels: Boston, Denmark, New York, Pittsburgh; Denmark was used as the reference category), education (1—below high school, 0—otherwise), smoking (smoked > 100 cigarettes in lifetime: yes [1]/no [0]), medication use (anti-diabetic, lipid-lowering, anti-hypertensive) (1—used, 0—did not use), fasting (1—≥ 8 h, 0—otherwise), and an 85-item deficits index (DI) (20). The DI (also known as a frailty index) aggregates a number of various health traits into a single measure and it is computed as the number of failed or abnormal traits (or “deficits”) divided by the total number of traits measured in individual at respective age (21, 22). An important advantage of the DI is that it can be constructed using the set of variables available in a specific dataset as its properties are weakly sensitive to the selection of a specific set of variables as shown in different studies [see e.g., (23–26)]. To construct the DI in the LLFS, we used health-related variables collected in LLFS that cover major health dimensions such as disability, cognition, morbidities, depression, physical performance, etc. Dichotomous variables were recoded as 1—deficit; 0—no deficit. Non-dichotomous variables were recoded as outlined in Kulminski et al. (20). The list of 85 variables used in the DI is provided in Table 1 in Kulminski et al. (20). The DI is constructed as a sum of the recoded variables divided by the number of variables measured in the respective individual. We computed receiver operating characteristic (ROC) curves and areas under the ROC curves (AUC) in logistic regression models with binary indicator of death (1—died during the follow-up, 0—alive) as the outcome for four combinations of D_M_ and DI variables (both D_M_ and DI, DI only, D_M_ only, none) used as covariates. All models were adjusted for other covariates specified above (sex, field center, education, smoking, medication use, fasting, and age). We did these calculations in the entire sample and also performed analyses stratified by generation and spouse status. Leave-one-out cross-validation was used for model evaluation in all calculations. See also description of additional computations in section Sensitivity Analyses.

Statistical analyses, data preparation, and visualization were done in SAS 9.4 (SAS/STAT 14.3) and R 3.5.0.

### Sensitivity Analyses

We performed sensitivity analyses to check whether our conclusions are sensitive to different aspects of the computation procedures, which might hypothetically affect the results. First, we considered different sets of biomarkers in computations of D_M_. We added the biomarkers used in our previous applications of D_M_ in the Framingham Heart Study (7, 12) to the list of the original 19 biomarkers from Sebastiani et al. (19). We also created D_M_ variants focusing on the subsets of biomarkers with absolute values of correlations with age exceeding specific thresholds (0.05, 0.1, 0.15, 0.2) and removing highly correlated biomarkers (one of a pair of biomarkers with absolute value of correlation between the biomarkers exceeding 0.8). We also computed separate D_M_ variants selecting biomarkers positively and negatively correlated with age. Second, we estimated the models using the original (non-transformed) values of D_M_. Third, we repeated the analyses focusing on the subsample of whites (which constitute the majority of the LLFS sample, 99%). Fourth, we modified the method of computation of the reference population changing the threshold (<65 and <70 years) and also computing means and variance/covariance matrices separately in the US and Danish subsamples. We also repeated computations excluding individuals with prevalent diseases (cancer, CVD, diabetes, AD or dementia) at the baseline [to focus on healthier reference populations, as discussed in (10)] and/or spouses (as the spousal groups are relatively small and spouses may also tend to share health habits). Fifth, we followed the common practice in the DI literature [e.g., (27)] and calculated DI only for individuals in whom <20% of the respective variables were missing. Sixth, we repeated the analyses using multiple imputation (MI) for biomarkers and other covariates with missing data (see notes under Table 1). We performed MI using the R-package *mice* (28) and SAS/STAT PROC MI/MIANALYZE (as needed for different analyses) in two scenarios: (a) we imputed (Box-Cox transformed) individual biomarkers under the assumption of multivariate normality using respective approaches (the joint modeling in *mice*, MCMC in SAS); (b) we imputed both (Box-Cox transformed) individual biomarkers and other covariates with missing values (education, smoking, anti-diabetic drugs, anti-hypertensive drugs, lipid-lowering drugs, fasting; see notes under Table 1) using fully conditional specification (29). The results using both approaches were similar; therefore, we report only the latter approach. We generated 25 datasets with imputed values of biomarkers and other covariates and computed D_M_ in each dataset using the observed and imputed data. Then we repeated the Cox model and the ROC/AUC analyses in each dataset and pooled respective estimates (the regression parameter estimates and differences between AUCs) using the standard tools implemented in the software to make statistical inference from imputed data. Seventh, we estimated the Cox model with D_M_ included as a categorical variable quantifying the quartiles of D_M_ with the first quartile as a reference category (see note under Table 2 about the proportionality of hazards assumption). Eighth, we recalculated the ROC/AUC analyses taking into account the relatedness between individuals (probands, their siblings, and offspring) using SAS/STAT PROC GENMOD. The results were nearly identical to those from SAS/STAT PROC LOGISTIC which did not make such adjustments; therefore, only the latter are reported in the text. Ninth, we repeated computations excluding individuals who died within a short period of time (one and 2 years) since the baseline to focus on predicting more distant events (considering the hypothetical possibility of reverse causation in cases when deaths occurred shortly after the measurements of biomarkers).

**Table 2:** Results of the Cox proportional hazards model applied to D_M_ at the baseline LLFS visit and follow-up mortality.

**Generation**	**Spouse status**	***N***	***N* deaths**	**Coef**.	**SE of Coef**.	***P*-value**	**HR**	**95% CI**	***SD* of D_**M**_**
Probands	Non-spouses	885	654	2.16	0.38	9.72 × 10^−12^	1.30	[1.21, 1.41]	0.12
	Spouses	128	67	4.23	1.39	1.98 × 10^−3^	1.75	[1.23, 2.50]	0.13
	All	1,013	721	2.22	0.36	4.29 × 10^−13^	1.32	[1.23, 1.42]	0.13
Offspring	Non-spouses	1,682	127	2.31	0.66	9.49 × 10^−5^	1.40	[1.18, 1.66]	0.15
	Spouses	565	50	1.41	1.04	0.27	1.22	[0.86, 1.74]	0.14
	All	2,247	177	2.09	0.55	1.72 × 10^−4^	1.35	[1.16, 1.58]	0.14
All	Non-spouses	2,567	781	2.18	0.32	1.89 × 10^−14^	1.42	[1.30, 1.55]	0.16
	Spouses	693	117	2.12	0.78	0.02	1.36	[1.05, 1.77]	0.15
	All	3,260	898	2.22	0.30	3.84 × 10^−16^	1.42	[1.30, 1.54]	0.16

*(1) The table displays sample sizes (N), numbers of deaths (N Deaths), estimates of the regression coefficient for the D_M_ variable in the Cox model (Coef.), their standard errors (SE of Coef.), p-values for the null hypothesis Coef. = 0 (P-value), and hazard ratios (HR) per standard deviation of D_M_ (SD of D_M_) along with their 95% confidence intervals (95% CI). (2) Respective quantities are shown for probands' generation (Generation = “Probands”; includes probands and their siblings enrolled in LLFS), offspring generation (Generation = “Offspring”; includes offspring of probands and their siblings enrolled in LLFS) and combined sample of probands' and offspring generations (Generation = “All”). Within each generation, the results are shown by spouse status: Spouse Status = “Non-spouses” includes probands and siblings, their offspring, and both, in the respective generations; Spouse Status = “Spouses” includes spouses of probands and siblings, their offspring, and both, in the respective generations; Spouse Status = “All” includes both spouses and non-spouses from the respective generations. (3) Proportionality of hazards assumption was tested using the test based on Schoenfeld residuals implemented in the R package “survival.” Respective p-values were larger than 0.05 (indicating that the use of the proportional hazards model was justified) for all cases displayed in the table except for Generation = “Probands” and Spouse Status = “All” (p = 0.045) (see Sensitivity Analyses)*.

## Results

### Descriptive Analyses

Table 1 shows the characteristics of the LLFS sample at the baseline visit including information on the 19 biomarkers used in construction of D_M_. See notes under the table for the number of missing values for each variable. The table indicates that participants from the probands' generation are about 23–30 years older in average than participants from the offspring generation. The proportion of females in the “Probands' Spouses” group is higher than in the other groups (possibly because females have better survival than males so that female spouses have higher chances to be included in this group). The proportion of participants from US field centers is higher in the probands' generation reflecting the sample recruitment specifics of the study. There are differences in proportions of low educated participants and smokers between the probands' and offspring generations that reflect the cohort/time trends in education and smoking patterns in the contemporary populations. Medication use also differs between the generations and it is more prevalent in the older groups (the probands' generation). All differences between the groups for the characteristics described above are significant (*p* = 0.0003 for lipid-lowering medication use; *p* < 0.0001 for all other) except for anti-diabetic medication use (*p* = 0.07).

The follow-up period since the baseline is relatively long in this study [e.g., the mean follow-up period for alive participants in LLFS is similar to the mean follow up in the Women's Health and Aging Study used in (2)]. As expected, the mean follow-up period is larger in the younger groups (the offspring generation) and the mean time until death is smaller in the older groups (the probands' generation). Also expectedly, the mean follow-up time for those who survived is larger than the mean time until death for those who died, in all groups (*p* < 0.0001 in all cases described above).

Participants from the older groups (the probands' generation) had higher prevalence of major diseases (cancer, CVD, diabetes, and AD or dementia; see section Data; *p*-values for differences between the groups: *p* < 0.0001 for cancer, CVD, and AD or dementia, *p* = 0.0003 for diabetes). However, differences in incidence of new cases of these diseases did not follow the uniform pattern. While the proportions of new cases of CVD and AD or dementia were higher in the probands' generation, the proportions of new cancer cases did not differ substantially between the groups and the proportions of new diabetes cases tended to be higher in the offspring generation. All differences between the groups were significant (*p* < 0.0001) except for cancer incidence (*p* = 0.5).

Table 1 also presents descriptive statistics for the 19 biomarkers used in computations of D_M_. We note that the biomarkers selected for this study were those from Sebastiani et al. (19) which were found to change with age in that study. Accordingly, all these biomarkers were highly correlated with age (*p* < 0.0001) in our analysis and their mean values changed, respectively, in the older and younger groups (*p* < 0.0001). We note however, that these results are purely descriptive and do not explore how multiple factors (except age) may contribute to such differences (or the absence of those) between the groups. We take into account appropriate variables in the regression analyses presented in the next sections.

**Figure 1:**
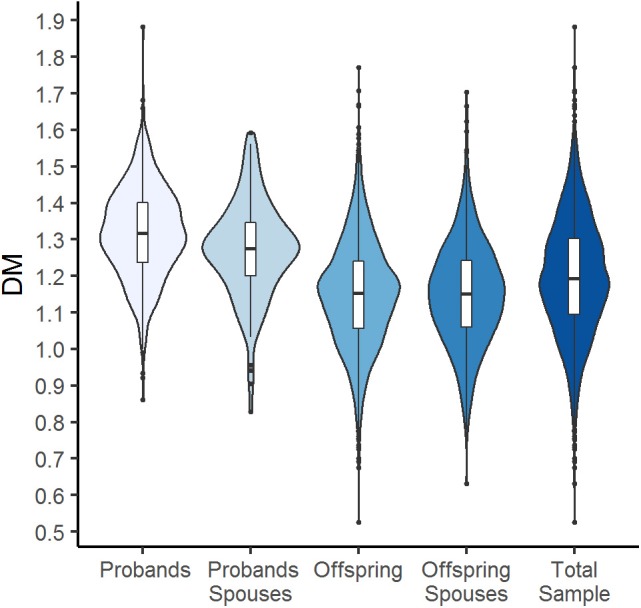
Violin plots with box plots showing D_M_ for the total sample and by generation and spouse groups. The blue-colored shapes represent a kernel density plot of the distribution of D_M_. Line, box, and points represent median, interquartile range (IQR), and outliers that are outside of 1.5 times the IQR.

Figure 1 shows violin plots for D_M_ distribution in the total sample and by groups. As one would expect (considering the fact that the offspring generation is much younger than the probands' one), D_M_ distributions differ substantially between the generation groups and participants from the probands' generation show a higher level of dysregulation (that is, larger D_M_) compared to offspring.

**Figure 2:**
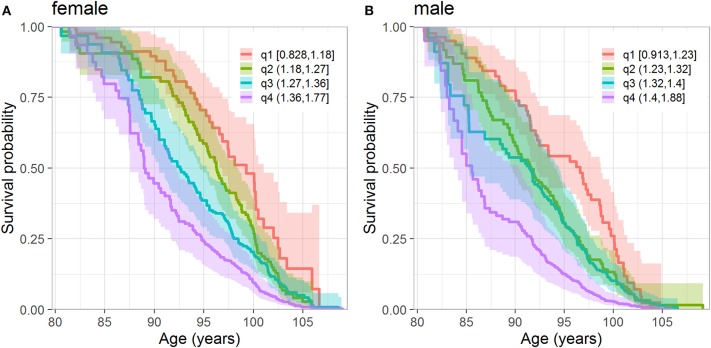
Kaplan-Meier estimates of conditional survival function of females **(A)** and males **(B)** according to the quartiles of D_M_. **(A)** Quartiles are calculated from females who survived until 80 years. **(B)** Quartiles are calculated from males who survived until 80 years. The numbers in the legend denote values of D_M_ in respective quartiles. The dark lines denote the point estimates of the survival functions and lighter colored areas denote their 95% confidence intervals.

We also investigated whether the level of physiological dysregulation (D_M_ values) at the baseline differentiates individuals according to their subsequent survival chances. Figure 2 displays the Kaplan-Meier estimates of the survival functions (conditional at age 80 years) for the strata defined by the quartiles of D_M_ distribution (computed separately for females and males). The figure shows that females and males with the lowest level of dysregulation (i.e., the first quartile of D_M_) have the best survival chances whereas those with the highest level of dysregulation (i.e., the fourth quartile of D_M_) have the worst survival, and those from the middle quartiles are in between these two extremes. Figure S1 shows the same curves for the combined sample of females and males. We note that such figures may provide some simple empirical evidence about the relationship between D_M_ and mortality; however, additional analyses are needed to take into account relevant factors (covariates) that can confound the observed association of D_M_ with mortality. Such analyses are presented in the next section.

### Association of D_M_ With Mortality

Table 2 displays the results of the Cox proportional hazards model applied to data on D_M_ constructed from biomarkers measured at the baseline visit and follow-up information on mortality in LLFS participants (total sample and stratified analyses by generation and/or spouse status, see notes under the table). Analyses of the total LLFS sample showed that higher D_M_ values are associated with higher mortality risk: hazard ratio (HR) per standard deviation (SD) of D_M_ is 1.42 (95% confidence interval, CI: [1.30, 1.54]). Similar associations were observed in strata by generation and/or spouse with HRs per SD of D_M_ ranging from 1.22 to 1.75 (however, the results were non-significant for spouses in the offspring generation which had the smallest number of deaths among all strata).

### Predictive Performance Analyses

**Figure 3:**
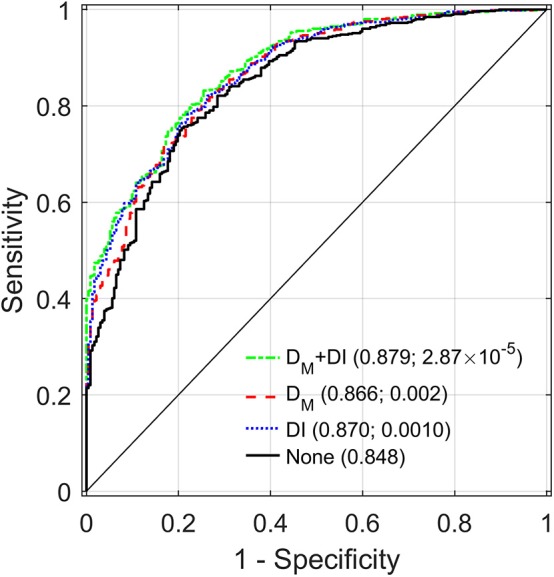
Receiver operating characteristic (ROC) curves for different models applied to a sample of LLFS probands and their siblings. “D_M_+DI” displays the ROC curve for the model with D_M_ also adjusted for DI and other relevant covariates (see Materials and Methods). “D_M_” (“DI”) corresponds to the ROC for the model with D_M_ (DI) and the other covariates. “None” shows the ROC for the reference model including only the other covariates but not D_M_ and DI. Areas under the ROC curves (AUC) and *p*-values for the null hypotheses about a zero difference between AUCs in the respective model and the reference model (“None”) are presented in parentheses.

Table 3 compares the performance of different models in predictions of mortality during the follow-up in LLFS, for the total sample and in the strata (same as in Table 2). The table shows the estimates of the areas under the receiver operating characteristic curves (AUC) for the reference model which includes age and other covariates (see section Association of D_M_ with Mortality and Predictive Performance Analyses) but does not include D_M_ and DI and the estimates of AUC in the models with D_M_ and/or DI (along with age and other covariates) and differences between AUC (dAUC) in these models and the reference model. The analyses indicated that addition of D_M_ and/or DI significantly improves the predictive performance of the models compared to the reference model in the total sample (*p*-values for the null hypothesis dAUC = 0 are 1.5 × 10^−5^, 4.05 × 10^−3^, and 9.19 × 10^−5^, for the models with D_M_+DI, D_M_, and DI, respectively). Analyses in generation and spouse status strata revealed that the largest increase in AUC was observed for non-spouses from probands' generation in the model including D_M_ and DI (dAUC = 0.032, *p* = 2.87 × 10^−5^). Similarly, for the model with D_M_, the largest increase in AUC was observed in the same stratum. The models with DI (based on 85 health-related deficits) and D_M_ (based on 19 biomarkers) produced similar dAUC's in this case. Figure 3 displays the AUCs for all four models in this stratum. Also we observed that in some cases (the offspring generation in the model with D_M_ and spouses in each generation in all models) differences between AUCs in the reference model and in the models with D_M_ and or DI did not reach statistical significance.

**Table 3:** Performance of different models in predictions of mortality during the follow-up in LLFS.

**Model**	**Generation**	**Spouse status**	***N***	***N* deaths**	**AUC**	**dAUC**	**SE of dAUC**	**95% CI of dAUC**	***P*-value**
D_M_+DI	Probands	Non-spouses	886	654	0.879	0.032	0.008	[0.017, 0.046]	2.87 × 10^−5^
		Spouses	128	67	0.751	0.017	0.023	[−0.028, 0.061]	0.46
		All	1,014	721	0.876	0.027	0.007	[0.014, 0.041]	8.60 × 10^−5^
	Offspring	Non-spouses	1,691	127	0.785	0.025	0.012	[4.1 × 10^−4^, 0.049]	0.046
		Spouses	574	50	0.820	0.019	0.015	[−0.012, 0.049]	0.23
		All	2,265	177	0.802	0.025	0.010	[0.005, 0.044]	0.01
	All	Non-spouses	2,577	781	0.939	0.008	0.002	[0.003, 0.012]	0.0003
		Spouses	702	117	0.890	0.021	0.008	[0.005, 0.037]	0.009
		All	3,279	898	0.934	0.009	0.002	[0.005, 0.013]	1.50 × 10^−5^
D_M_	Probands	Non-spouses	886	654	0.866	0.018	0.006	[0.006, 0.029]	0.002
		Spouses	128	67	0.738	0.003	0.019	[−0.033, 0.040]	0.86
		All	1,014	721	0.866	0.016	0.005	[0.006, 0.027]	0.002
	Offspring	Non-spouses	1,691	127	0.768	0.008	0.008	[−0.008, 0.024]	0.34
		Spouses	574	50	0.805	0.004	0.009	[−0.013, 0.021]	0.61
		All	2,265	177	0.784	0.007	0.006	[−0.006, 0.019]	0.30
	All	Non-spouses	2,577	781	0.935	0.004	0.001	[0.001, 0.007]	0.01
		Spouses	702	117	0.877	0.008	0.005	[−0.002, 0.018]	0.11
		All	3,279	898	0.929	0.004	0.001	[0.001, 0.007]	0.004
DI	Probands	Non-spouses	886	654	0.870	0.022	0.007	[0.009, 0.035]	0.0010
		Spouses	128	67	0.752	0.017	0.018	[−0.017, 0.052]	0.33
		All	1,014	721	0.868	0.019	0.006	[0.007, 0.031]	0.002
	Offspring	Non-spouses	1,691	127	0.780	0.019	0.010	[−0.001, 0.039]	0.07
		Spouses	574	50	0.821	0.020	0.016	[−0.011, 0.051]	0.20
		All	2,265	177	0.798	0.021	0.009	[0.003, 0.038]	0.02
	All	Non-spouses	2,577	781	0.937	0.005	0.002	[0.002, 0.009]	0.001
		Spouses	702	117	0.888	0.019	0.008	[0.004, 0.035]	0.01
		All	3,279	898	0.932	0.007	0.002	[0.003, 0.010]	9.19 × 10^−5^
None	Probands	Non-spouses	886	654	0.848				
(reference)		Spouses	128	67	0.735				
		All	1,014	721	0.849				
	Offspring	Non-spouses	1,691	127	0.761				
		Spouses	574	50	0.801				
		All	2,265	177	0.778				
	All	Non-spouses	2,577	781	0.931				
		Spouses	702	117	0.869				
		All	3,279	898	0.925				

*(1) The table shows sample sizes (N), numbers of deaths (N Deaths), estimates of the areas under the receiver operating characteristic curves (AUC), their differences from AUC in the reference model (dAUC), standard errors (SE of dAUC) and 95% confidence intervals (95% CI of dAUC) of the differences, and p-values for the null hypothesis dAUC = 0 (P-value). (2) See notes to Table 2 regarding columns Generation and Spouse Status. (3) The rows for Model = “D_M_+DI” display the results for the model with D_M_ also adjusted for DI and other relevant covariates (see Materials and Methods) vs. the reference model including only the other covariates but not D_M_ and DI [Model = “None (reference)”]. The rows for Model = “D_M_” (Model = “DI”) correspond to the results for the model with D_M_ (DI) and the other covariates vs. the reference model. (4) Differences between N in this table and Table 2 are because Cox models removed individuals whose age at baseline was equal to age at censoring (19 individuals were lost to follow-up right after visit 1 so that their age at censoring was set to age at baseline)*.

### Sensitivity Analyses

We ran different sensitivity analyses (see section Sensitivity Analyses) in the total sample, which confirmed the observations, reported above. Specifically: (1) We ran the Cox and logistic regression (AUC) analyses for D_M_'s constructed from different sets of biomarkers (see section Sensitivity Analyses) which confirmed the associations of D_M_ with mortality (HR ranging from 1.26 to 1.44; *p*-values ranging from 2.42 × 10^−21^ to 8.41 × 10^−11^; here and below HRs are per SD of D_M_) and that addition of D_M_ significantly improves the predictive performance of the models (dAUCs range from 0.002 to 0.004 and *p*-values range from 5.73 × 10^−4^ to 3.63 × 10^−2^ in the model with D_M_; dAUCs range from 0.008 to 0.009 and *p*-values range from 4.36 × 10^−7^ to 1.6 × 10^−4^ in the model with D_M_+DI). (2) We repeated the analyses with the original (non-transformed) values of D_M_ which showed similar results: HR = 1.3 (95% CI: [1.22, 1.38]); dAUC = 0.004 (*p* = 1.85 × 10^−3^) for the model with D_M_, dAUC = 0.009 (*p* = 5.75 × 10^−6^) for the model with D_M_+DI. (3) We computed D_M_ and ran analyses in the subsample of whites which resulted in comparable estimates: HR = 1.42 (95% CI: [1.31, 1.55]); dAUC = 0.004 (*p* = 3.83 × 10^−3^) for the model with D_M_, dAUC = 0.009 (*p* = 1.48 × 10^−5^) for the model with D_M_+DI. (4) We confirmed that the observations are not sensitive to the choice of the reference population (threshold for reference population 65 and 70 years, and means and variance/covariance matrices computed separately in the US and Danish subsamples): HR range from 1.36 to 1.41 and *p*-values range from 7.43 × 10^−16^ to 7.72 × 10^−15^ in the Cox analyses; dAUCs range from 0.003 to 0.004 (from 0.008 to 0.009) and *p*-values range from 3.18 × 10^−3^ to 9.1 × 10^−3^ (from 1.2 × 10^−5^ to 3.62 × 10^−5^) in the model with D_M_ (D_M_+DI). We also found that the results do not change substantially when the reference population excludes unhealthy individuals (see section Sensitivity Analyses) and/or spouses. For example, for the total sample, HR ranged from 1.41 to 1.45 (*p*-values: from 3.72 × 10^−17^ to 5.30 × 10^−16^) in the Cox analyses; dAUCs ranged from 0.0040 to 0.0045 (from 0.0086 to 0.0090) and *p*-values ranged from 2.04 × 10^−3^ to 4.05 × 10^−3^ (from 7.97 × 10^−6^ to 1.34 × 10^−5^) in the model with D_M_ (D_M_+DI). (5) We observed that adjusting the approach to compute DI (calculating it only for individuals in whom < 20% of the respective variables were missing) did not substantially affect the results: HR = 1.43 (95% CI: [1.32, 1.56]); dAUC = 0.004 (*p* = 5.97 × 10^−3^) for the model with DI, dAUC = 0.009 (*p* = 1.8 × 10^−5^) for the model with D_M_+DI. (6) We repeated the analyses using multiple imputation which replicated the reported findings: HR = 1.38 (95% CI: [1.27, 1.49]); dAUC = 0.003 (*p* = 1.43 × 10^−3^) for the model with D_M_, dAUC=0.007 (*p* = 1.37 × 10^−7^) for the model with D_M_+DI. (7) We repeated the Cox regression analyses in the probands' generation with D_M_ included as a categorical variable that confirmed the association with mortality (HR for 3rd quartile vs. 1st quartile of D_M_: 1.84 [1.28, 2.65]; HR for 4th quartile vs. 1st quartile: 2.38 [1.66, 3.42]) and also addressed the issue with the proportionality of hazards assumption in this stratum (*p* = 0.47). (8) We ran analyses of the total sample removing individuals dying within a short time interval (one and 2 years) since baseline. The analyses confirmed the associations of D_M_ with mortality: HR = 1.4 (95% CI: [1.28, 1.52]) for a 1-year interval and HR = 1.3 (95% CI: [1.21, 1.45]) for a 2-years interval. We also found that the conclusions that addition of D_M_ significantly improves the predictive performance of the models still holds in such cases: dAUC = 0.004 (*p* = 5.81 × 10^−3^) for the model with D_M_, dAUC = 0.009 (*p* = 1.88 × 10^−5^) for the model with D_M_+DI, for a 1-year interval; dAUC = 0.004 (*p* = 1.02 × 10^−2^) for the model with D_M_, dAUC = 0.009 (*p* = 3.73 × 10^−5^) for the model with D_M_+DI, for a 2-years interval.

In sum, our extensive sensitivity analyses confirmed that our conclusions are not sensitive to different aspects of the computation procedures.

## Discussion

In this paper, we confirmed that the composite measure of physiological dysregulation (D_M_) is associated with mortality in LLFS (with larger D_M_ associated with increased mortality risk), similar to other studies (2, 7, 9, 12). We showed also that addition of D_M_ significantly improves mortality predictions compared to the reference model (containing age, sex, and other relevant covariates) in the total LLFS sample. We also found that the largest improvement in predictive performance when adding D_M_ to the predictive model [with or without another composite index, DI, (20)] is observed in the proband's generation, that is, for individuals from the families selected for exceptional longevity. The LLFS participants from such families constitute by design a very selected sample from the general population [e.g., only about 2.2% of participants from the Utah Population Database (30) would be enrolled in the LLFS according to its criteria] and the LLFS participants have much better survival chances than their age peers from a general population (14). The present work is the first study that explored the association of D_M_ with mortality and its predictive performance in such a unique sample. As we showed in Yashin et al. (14), the improved predictions of lifespans based on applications of the deficit index (DI) (20) resulted in detection of additional signals in genome-wide association studies (GWAS) of longevity which were not observable in GWAS with actual ages at death of deceased individuals. Importantly, we showed that the benefits of using predicted vs. observed lifespan data in the GWAS of human longevity are most noticeable for shorter follow-up periods, which is the case for many contemporary studies collecting genetic data, including LLFS. As the results of the present work indicate, including D_M_ in predictive models can provide further benefits for GWAS of human longevity. There are additional opportunities for improving the power of such studies if appropriate methods are used (31).

As other studies showed, D_M_ is associated not only with mortality but also with other health and aging related outcomes (7, 9, 11, 32). In particular, as discussed in our recent study (7), D_M_ can be a promising indicator of declining robustness and resilience during aging, and may precede clinical manifestation of not just one but many diseases even in the absence of strong clinical diagnostic markers pointing out to a specific pathology. Given that, D_M_ could be an especially useful predictor of mortality among the elderly without major chronic diseases. In Arbeev et al. (12), we implemented D_M_ in the framework of the stochastic process model (SPM) of aging (33), which allowed us to observe regularities in dynamic characteristics of trajectories of D_M_ in relation to different aging-related characteristics such as decline in stress resistance and adaptive capacity, and to evaluate how such characteristics might be associated with an increase in mortality risk with age. The LLFS provides opportunities to perform similar analyses in a unique sample of individuals from families enriched for exceptional survival who not only have better survival (14) but also have better health and functioning (15) than a general population. Applications of SPM to analyses of D_M_ in this unique sample and comparison with other studies can help reveal which particular aging-related characteristics differ in individuals with exceptional health and lifespan compared to average individuals and how these differences can propagate to the observed differences in morbidity and mortality risks. Applications of this model will also provide opportunities to take into account varying strength of association of biomarkers with mortality at different ages in construction of the composite measures. In addition, the SPM versions developed for analyses of genetic data (34, 35) can be applied to find genetic factors associated with various hidden aging-related mechanisms (e.g., decline in adaptive capacity and stress resistance, allostatic adaptation) which are not directly observed in the data but can be estimated by this model using longitudinal measurements of biomarkers and follow-up data on mortality or morbidity.

In addition to composite biomarkers such as D_M_, other approaches were suggested in the literature to quantify biological aging, which can shed light on different aspects of the aging process (32, 36). The upcoming collection of extensive omics information (whole genome sequencing, methylomics, transcriptomics, metabolomics, proteomics) in the LLFS participants should open new perspectives for comprehensive evaluation of potential biological mechanisms and pathways related to exceptional longevity and delayed aging in this unique sample. We note also that these future studies need to be accompanied by relevant methodological developments that would take into account specifics of the data (e.g., informative missingness, multi-generational sample, longitudinal omics profiles) to generate valid statistical inferences.

This study has several limitations. We analyzed a unique sample (LLFS) which was selected for exceptional longevity (which was the goal of this study) and the LLFS participants also have better health and function in several domains compared to other cohorts (15). Therefore, the results are not generalizable to the general population. However, the association of D_M_ with mortality was already established in several other studies with health and survival patterns closer to a general population [e.g., (2, 7, 9, 12)]. The sample analyzed in our study is predominantly white (Table 1). Thus, applications to studies with sizable samples of other race and ethnic groups are necessary to confirm the results for such groups. As we do not have verified information on causes of death for deceased participants, we cannot exclude that some participants had non-natural causes of death (such as accidents, homicides, etc.) which are not related to D_M_. However, we note that most participants in our study are very old and these causes are not among the leading causes of death for such ages. In this study, we analyzed only one (baseline) measurement of D_M_. Even though the observed associations were still strong despite a relatively long follow-up period since baseline, future analyses of repeated measurements of biomarkers will allow investigating associations of dynamic characteristics of trajectories of cumulative biomarkers (such as D_M_) with mortality and exploring genetic underpinnings of such dynamics. This requires applying advanced statistical tools to appropriately handle methodological challenges in such analyses and this is a subject of our ongoing research.

Although this study was not performed in the clinical settings with patients' data, there is a potential for applications of D_M_ in such settings as we discussed in our prior research (7). Blood tests results and other relevant measures are routinely collected from patients and they can be used to construct D_M_ that can inform health practitioners about underlying transition to an unhealthy state even in the absence of specific pathological values of individual biomarkers. Also importantly, there is no “pre-defined” set of biomarkers that need to be included in such a measure. Therefore, it can be constructed from available biomarkers (e.g., standard laboratory tests) without incurring additional costs for data collection. As we showed in sensitivity analyses, the associations with mortality and improved predictive performance was observed for different subsets of biomarkers used in D_M_ (some of those sets were parsimonious ones with just a few biomarkers). Even though the concept of statistical distance measure computed from biomarkers showed its usefulness in several applications, this is still an active area of research. In particular, the approach to specify such a distance considering non-linear patterns of changes of many biomarkers with age is a subject of our ongoing research.

## Data Availability Statement

The Long Life Family Study (LLFS) data used in this study were provided by the LLFS Data Management and Coordinating Center (DMCC), Washington University, St. Louis: https://wustl.edu/. All questions regarding access to the LLFS data should be addressed to Professor Michael Province: mprovince@wustl.edu.

## Ethics Statement

The studies involving human participants were reviewed and approved by Duke Health IRB. The patients/participants provided their written informed consent to participate in this study.

## Author Contributions

KA conceived and designed the study, supervised statistical analyses and data preparations, and wrote the manuscript. OB prepared data, performed statistical analyses, and contributed to writing Materials and Methods section. HD and DW contributed to data preparation. SU, AK, ES, KC, MF, BT, JZ, and AY contributed to writing the manuscript. All authors read and approved the final manuscript.

### Conflict of Interest

The authors declare that the research was conducted in the absence of any commercial or financial relationships that could be construed as a potential conflict of interest.
