# Supplementary material for: Composite Measure of Physiological Dysregulation as a Predictor of Mortality: The Long Life Family Study
Source: Front Public Health. 2020 Mar 6;8:56. doi: 10.3389/fpubh.2020.00056 (PMC7067825; doi:10.3389/fpubh.2020.00056)
Supplement: Supplementary file 1 [file Data_Sheet_1.docx]

**Supplementary Materials**

**Table S1:** Sample characteristics of the reference population used in computations of the statistical distance measure (D_M_)

| **Sample Characteristics** | **Values** |
| --- | --- |
| Number of participants at baseline | 1361 |
| Number of deaths during the follow-up period | 31 |
| Age at baseline* | 53.8 ± 5.2 [24, 60] |
| Females (%) | 59.88 |
| Whites (%) | 99.04 |
| Participants from US field centers (%) | 70.32 |
| Low educated participants (below high school) (%) | 3.53 |
| Smokers (smoked >100 cigarettes in lifetime) (%) | 41.81 |
| Medication use: anti-diabetic (%) | 3.09 |
| Medication use: anti-hypertensive (%) | 18.15 |
| Medication use: lipid-lowering (%) | 15.43 |
| Fasting (>=8 hrs.) (%) | 92.8 |
| Follow-up period* | 9.7 ± 2.3 [0, 12.5] |
| Follow-up period for dead* | 6.2 ± 3.1 [1, 12.4] |
| Follow-up period for alive* | 9.8 ± 2.3 [0, 12.5] |
| Prevalence of cancer, N (%) | 134 (9.85) |
| Prevalence of CVD, N (%) | 43 (3.16) |
| Prevalence of AD or dementia, N (%) | 1 (0.07) |
| Prevalence of diabetes, N (%) | 47 (3.45) |
| Incidence of cancer, N (%) | 127 (9.33) |
| Incidence of CVD, N (%) | 38 (2.79) |
| Incidence of AD or dementia, N (%) | 1 (0.07) |
| Incidence of diabetes, N (%) | 37 (2.72) |
| Adiponectin, ng/mL (Adip)** | 10603 ± 5930 0.08 [1.7e-03] |
| Albumin, g/dL (Album)** | 4.1 ± 0.3 0.03 [0.25] |
| Absolute monocyte count, 10e9/L (Abs.M)** | 0.6 ± 0.2 8.3e-03 [0.76] |
| Creatinine, mg/dL (Creat)** | 1.0 ± 0.2 6.1e-03 [0.82] |
| Cystatin, mg/L (Cysc)** | 0.8 ± 0.2 0.16 [1.6e-09] |
| Dehydroepiandrosterone sulfate, ug/dL (DHEA)** | 105.4 ± 67.5 -0.21 [1.5e-14] |
| Hemoglobin, g/dL (Hgb)** | 14.2 ± 1.3 0.04 [0.16] |
| Glycosylated hemoglobin, % (HbA1c)** | 5.5 ± 0.5 0.15 [1.2e-08] |
| High-sensitivity C-reactive protein, mg/L (hsCRP)** | 2.4 ± 4.0 -0.04 [0.19] |
| Insulin-like growth factor 1, ng/mL (IGF1)** | 149.0 ± 61.2 -0.11 [2.8e-05] |
| Interleukin 6, pg/mL (IL-6)** | 1.2 ± 3.7 -0.03 [0.28] |
| Mean corpuscular volume, fl (MCV)** | 91.5 ± 5.1 0.04 [0.11] |
| N-terminal pro b-type natriuretic peptide, pg/mL (NT-proBNP)** | 64.5 ± 122.3 0.03 [0.30] |
| Red cell distribution width, % (RDW)** | 13.5 ± 1.0 0.03 [0.24] |
| Sex-hormone binding globulin, nmol/L (SHBG)** | 61.3 ± 37.2 -0.12 [1.7e-05] |
| Soluble receptor for advanced glycation endproduct, pg/mL (sRAGE)** | 520.0 ± 297.8 0.02 [0.45] |
| Total cholesterol, mg/dL (T.Chol)** | 207.0 ± 38.6 0.13 [3.1e-06] |
| Transferrin receptor, mg/L (Transf.R)** | 2.9 ± 1.0 -0.03 [0.27] |
| White blood cell count, 10e9/L (WBC)** | 5.8 ± 1.7 -0.01 [0.65] |

**Notes:** 1) ^*^: these rows display mean ± SD [range is shown in brackets]; 2) ^**^: these rows display mean ± SD and correlation with age [p-value for the null hypothesis on zero correlation is shown in brackets]


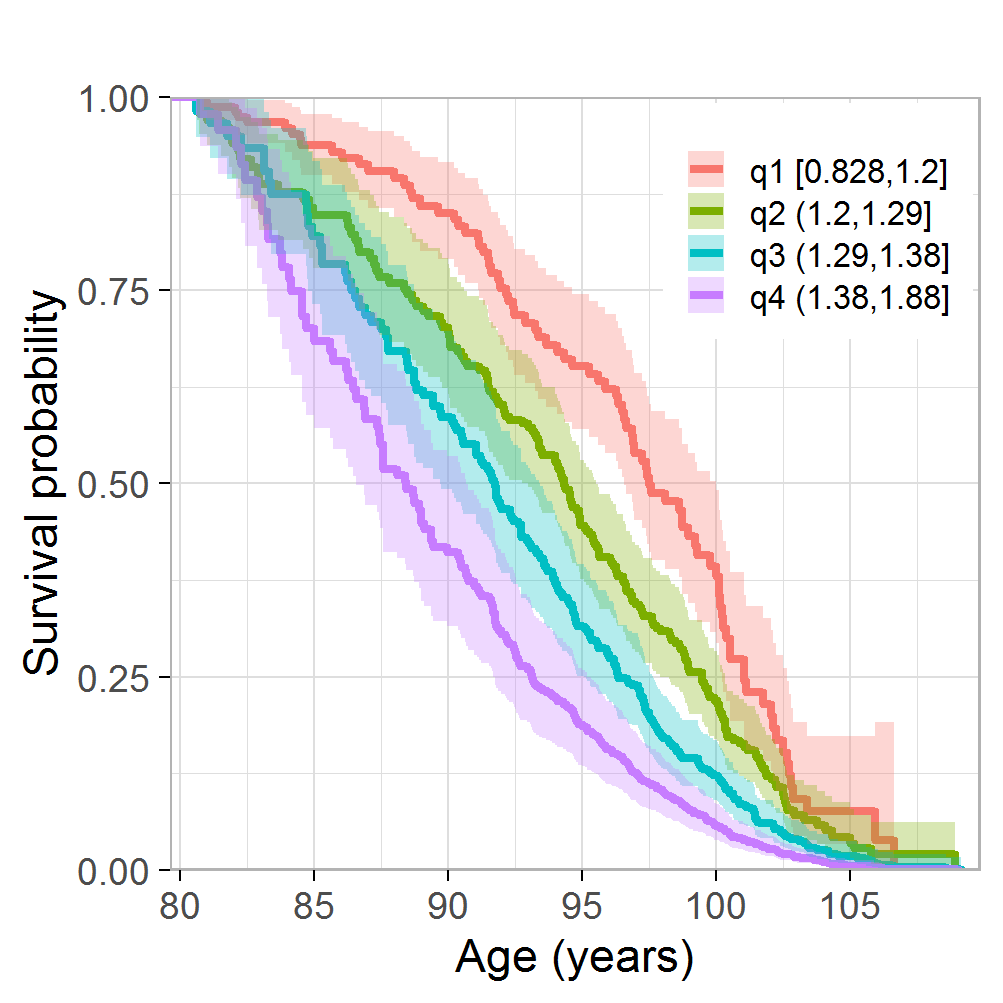


**Figure S1: Kaplan-Meier estimates of conditional survival function according to the quartiles of D_M_.** Quartiles are calculated from individuals who survived until 80 years. The numbers in the legend denote values of D_M_ in respective quartiles. The dark lines denote the point estimates of the survival functions and lighter colored areas denote their 95% confidence intervals.
